# Haptic assessment of bone quality in orthopedic surgery: no consensus but perspective for high training potential

**DOI:** 10.5116/ijme.5a38.168c

**Published:** 2017-12-22

**Authors:** Sven A.F. Tulner, Vilijam Zdravkovic, Fabrice Külling, Bernhard Jost, Gabor J. Puskas

**Affiliations:** 1 Department of Orthopaedics and Traumatology, Kantonsspital St. Gallen, Switzerland

**Keywords:** Haptic assessment, bone quality, orthopedic surgery, consensus, perspective, high training potential

## Introduction

The ability to intraoperatively assess bone quality is important for orthopedic surgeons because bone quality might be a decisive factor for treatment strategy.^1^ However, this ability is usually not systematically taught in the surgical curriculum. Orthopedic residents learn about the physical property of the bone early in their formation. They also learn about the radiological assessment of bone quality by computed tomography, measuring bone mineral density^2^ or by measuring the cortical thickness of the proximal humeral diaphysis in standard anterior-posterior X-rays or the deltoid tuberosity index.^3^^,^^4^ However, these preoperative radiological measurements are indirect assessments of bone quality that cannot dispense orthopedic surgeons from direct intraoperative assessment.

The bone quality of humeral metaphysis is crucial for the use of new stemless shoulder arthroplasty systems because the humeral component could loosen early (primary non-bonding) if press-fit fixation in the cancellous bone is not reliable.^5^ The decision if the bone shows "adequate" quality for using a particular implant design relies on the intraoperative judgment of the surgeon and depends on his manual skill and experience. Tools for intraoperative assessment of bone quality have been developed^6^^,^^7^ but are complicated and not readily available in a routine clinical setting, whereas simple tactile (haptic) assessment by the surgeon can easily be performed.

In surgical courses, young residents learn mainly operative techniques, especially the handling of surgical instruments and implants. Interestingly, however, assessment of bone quality by haptic perception is not systematically taught in the clinical training of orthopedics residents. It seems that surgeons individually develop a "feeling" about what is "adequate bone quality" by trial and error. However, we do not know if this feeling is the same for all orthopedic surgeons.

We believe that even in highly specialized and more technological medicine simple manual testing and haptic assessment remains crucial and might get lost in the contemporary education of young surgeons. Following questions arise: Can haptic assessment of bone quality regarding load-bearing capacity be standardized and systematically be taught and trained?

The purpose of this perspective, therefore, is to discuss the intraoperative haptic assessment of bone quality and to evaluate the precision of the assessment, its training and its learning process. This might open new questions and further directions in surgical education.

### Relation of haptic assessment and experience

We tested haptic assessment of bone quality in 41 staff members with different levels of experience. In a first step, they were asked to press on a pinch dynamometer as much as they believed normal cancellous bone must withstand without indentation corresponding to good bone quality. Regardless of the level of experience, there was a considerable divergence about how much pressure "good quality" bone should withstand. In a second step, participants could freely practice pressing a defined target force until they felt confident to reach it. This target force corresponded to the mean of the initially pressed force of all participants in step 1. As the third step, about seven days after the training, the participants had to press the target force. The 13 experienced surgeons were closest in pressing the target force. The difference of the test pressure to the target pressure was lower in expert group, when compared to the 18 less experienced surgeons or the 10 students. In addition, the distribution of the test pressure values was narrower in the expert group compared to the other two groups.

## Conclusions

With this perspective, we learned that surgeon's subjective haptic assessment of bone quality may significantly diverge and, what is more important, may not correspond to true bone quality. We do not know a threshold value of bone mineral density that would be relevant for decision making in stemless shoulder arthroplasty. Therefore, we could not use a particular bone quality threshold to be detected by haptic perception.

We realized that specific training of haptic assessment is inconsistent if not inexistent during the surgical education of young orthopedic surgeons and hypothesized that this might be the reason for this divergence. We experienced that our survey raised the awareness of our staff members about haptic assessment and experienced surgeons began to think how they could better transfer their knowledge to young colleagues. Since then residents were more often asked to touch and assess the bone physically and were not only taught about technical issues of surgery and asked to hold the surgical retractors. Knowing that haptic perception can be learned and trained we strongly believe that a biomechanical threshold of good cancellous bone quality could be defined and correlated to a corresponding target force for tactile pressure. We are now motivated to develop specific training models for haptic assessment of different bone qualities and are convinced that such training will increase the surgeon's intraoperative ability of a reliable uniform evaluation of bone quality and will lead to a consistent decision making during surgery. Especially young surgeons should be taught early in the career to train their abilities in haptic assessment, preferably in teaching and education centers prior to surgical exposure.

### Conflict of Interest

The authors declare that they have no conflict of interest.
